# Supplementary figures and images for: Evolutionary conservation and post-translational control of S-adenosyl-L-homocysteine hydrolase in land plants
Source: PLoS One. 2020 Jul 17;15(7):e0227466. doi: 10.1371/journal.pone.0227466 (PMC7367456; doi:10.1371/journal.pone.0227466)

Supplemental Figure 1

A)

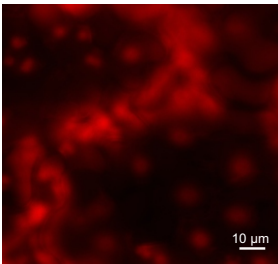

B)

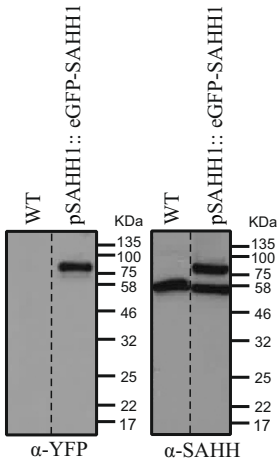

Supplement: S1 Fig — A) Confocal microscopy image obtained from A. thaliana wild type plant using microscopy settings for GFP imaging. The leaf was excited at 488 nm and fluorescence was detected at 493 to 598 nm wave length. Chlorophyll fluorescence was excited at 633 nm and detected at 647 to 721 nm wave length. The red color indicates chlorophyll autofluorescence. B) Immunoblots depicting EGFP-SAHH1 in A. thaliana wild type (WT) and a transgenic line stably expressing SAHH1p::EGFP-SAHH1. Proteins were separated on SDS-PAGE, and EGFP-SAHH1 was immunodetected with an anti-YFP antibody and SAHH was detected with an anti-SAHH antibody. (PDF) [file pone.0227466.s004.pdf]

Supplemental Figure 2

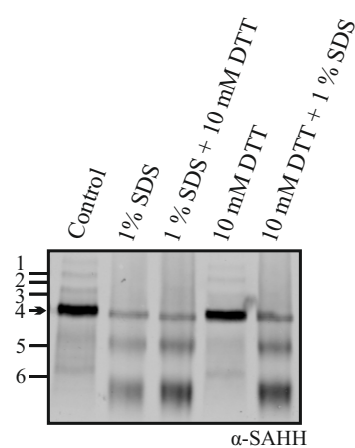

Supplement: S2 Fig — For combined treatments with SDS and DTT, the leaf extract was incubated in the presence of one chemical for 30 minutes, followed by addition of the other for 30 minutes. (PDF) [file pone.0227466.s005.pdf]

Supplemental Figure 3

A)

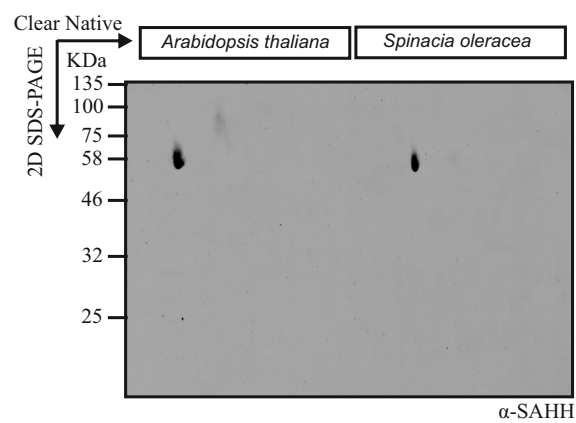

B)

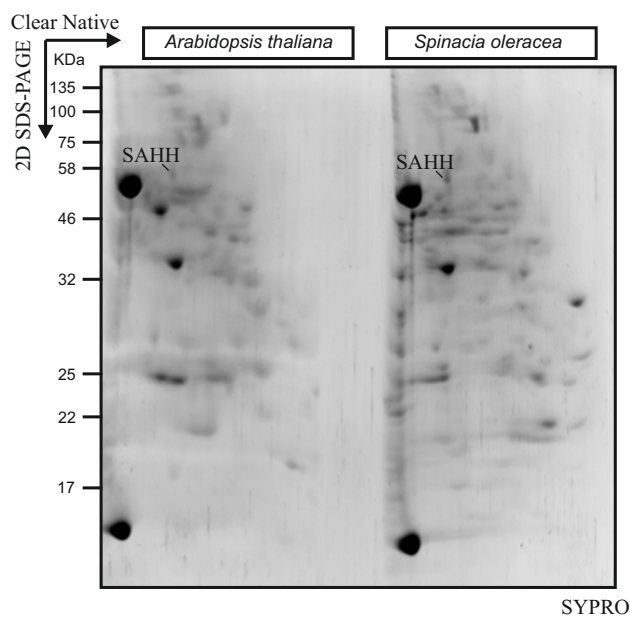

Supplement: S3 Fig — Protein complexes were separated CN-PAGE followed by 12% SDS-PAGE in the second dimension. A) Predominant protein spots as detected by immunoblot analysis using α-SAHH antibody. B) Total protein detection by SYPRO. The spots indicated as “SAHH” in A. thaliana and S. oleracea samples were excised from the gel and the presence of SAHH was confirmed by mass spectrometry as indicated in S3 Table. (PDF) [file pone.0227466.s006.pdf]

Supplemental Figure 4

\*Used in figure 5

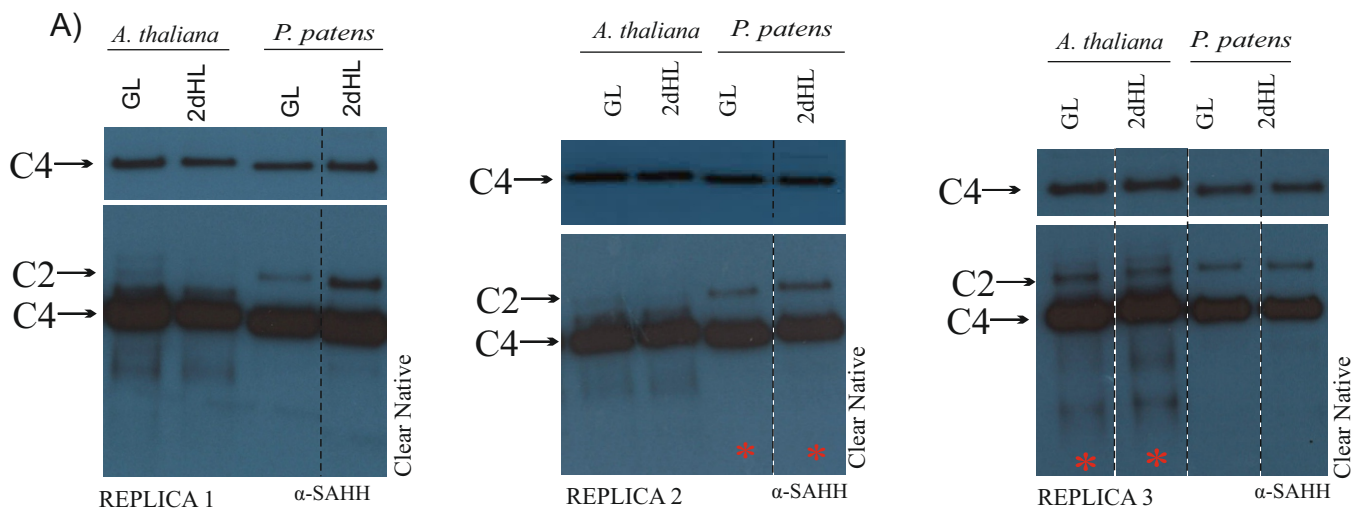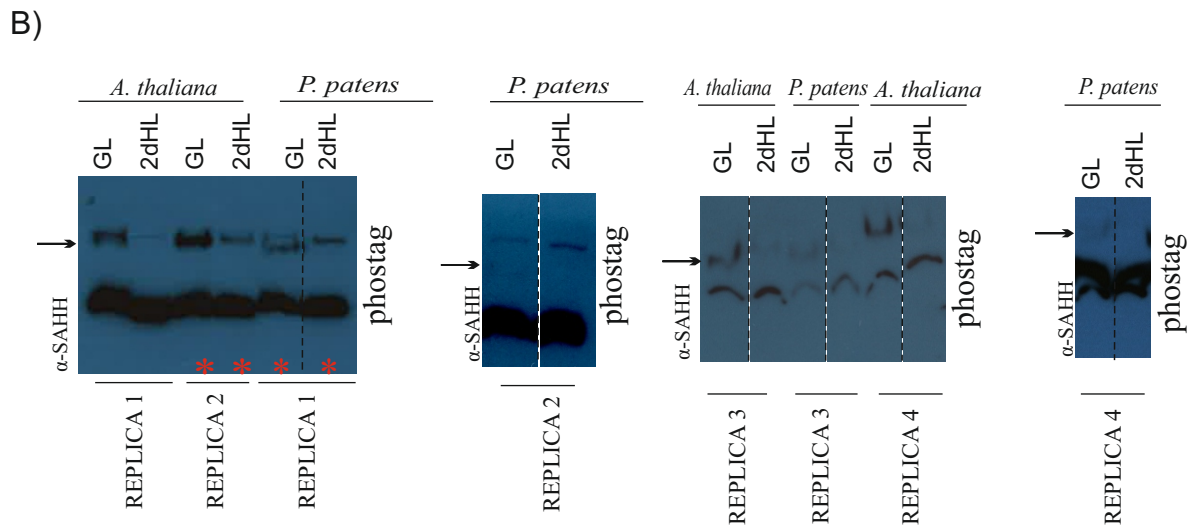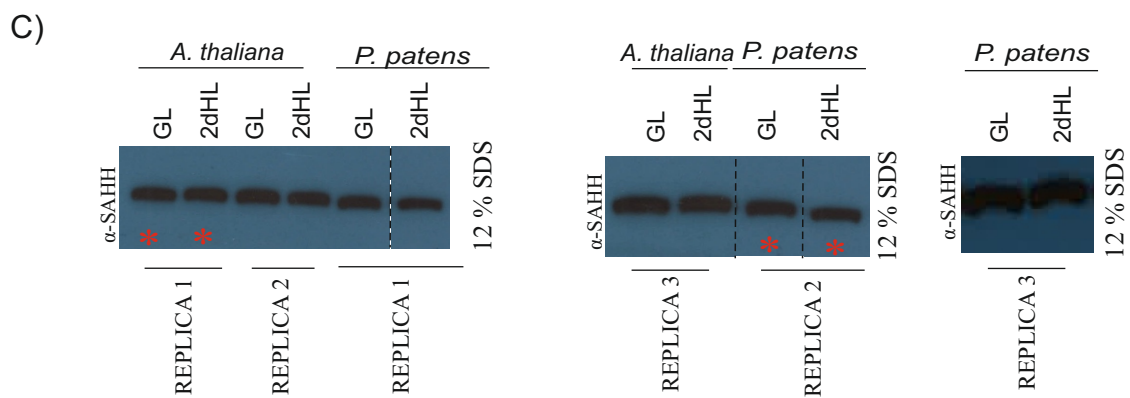

Supplement: S4 Fig — A. thaliana was grown under 130 μmol photons m-2 s-1 for 16 days and thereafter shifted 800 μmol photons m-2 s-1 for 2 days. P. patens was grown under 45 μmol photons m-2 sec-1 for 13 days and thereafter illuminated under 500 μmol photons m-2 s-1 for two days. The gel lanes indicated by asterisks were used to construct Fig 5. A) Oligomeric protein complexes as detected by anti-SAHH antibody and clear native (CN)-PAGE from three independent experiments. The upper panels depict immunoblots with a shorter exposure time required for visualization and quantification of the abundant SAHH complex 4. B) SAHH protein phosphorylation as detected by anti-SAHH antibody and Phostag-PAGE in A. thaliana and P. patens in growth light (GL) and after 2-day illumination under high light (2dHL). C) SAHH protein abundance as detected by anti-SAHH antibody and SDS-PAGE in A. thaliana and P. patens in growth light (GL) and after 2-day illumination under high light (2dHL). (PDF) [file pone.0227466.s007.pdf]

Figure 2

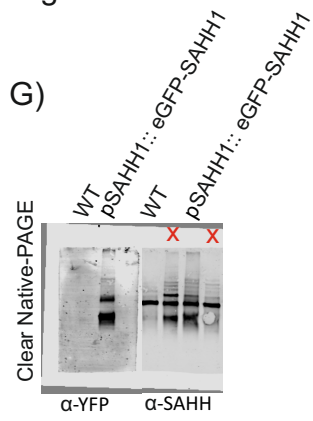

Figure 3

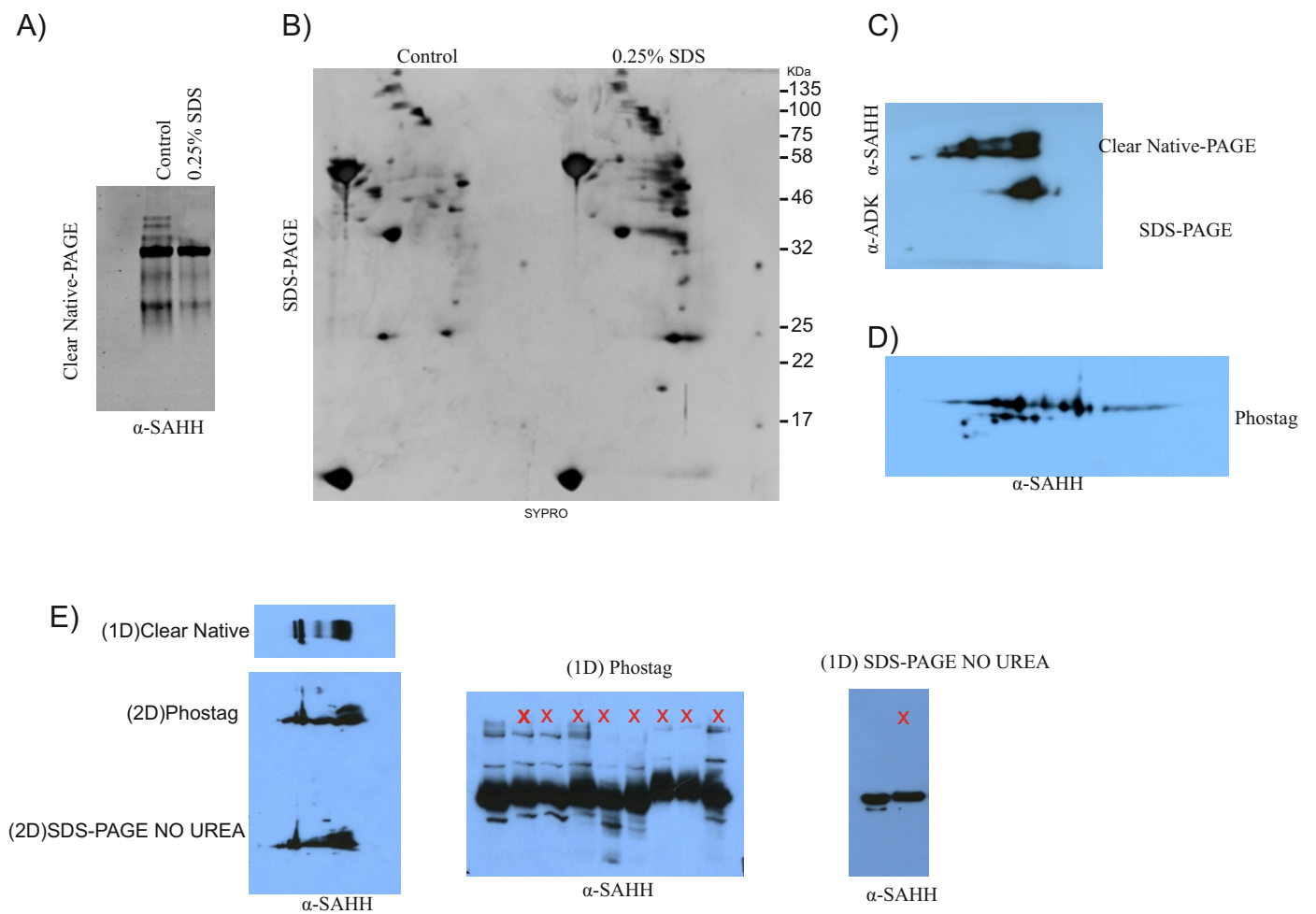

Figure 4 C

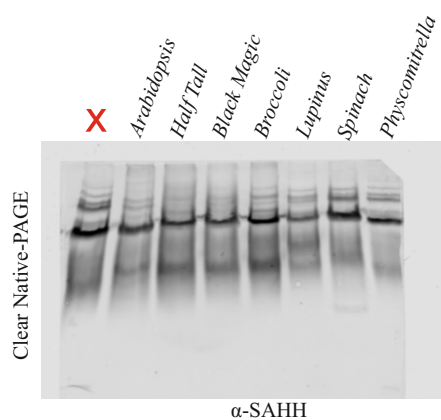

Figure 5

\* Used in figure 5

A)

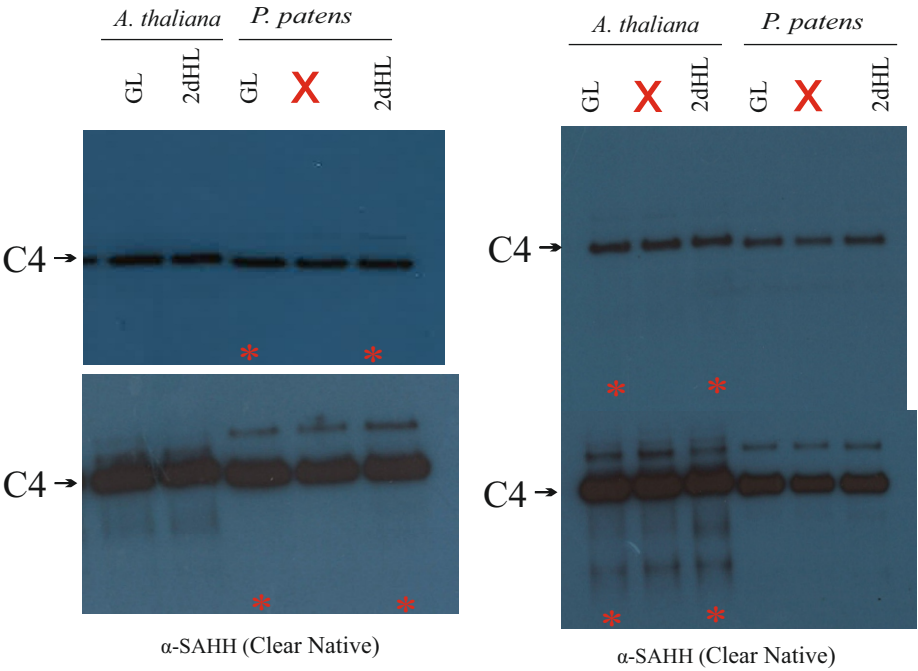

C)

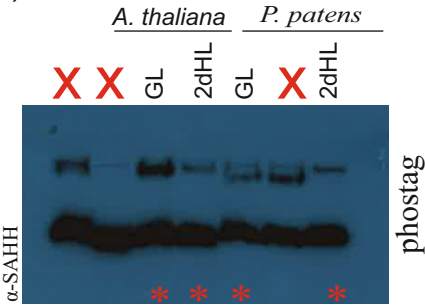

D)

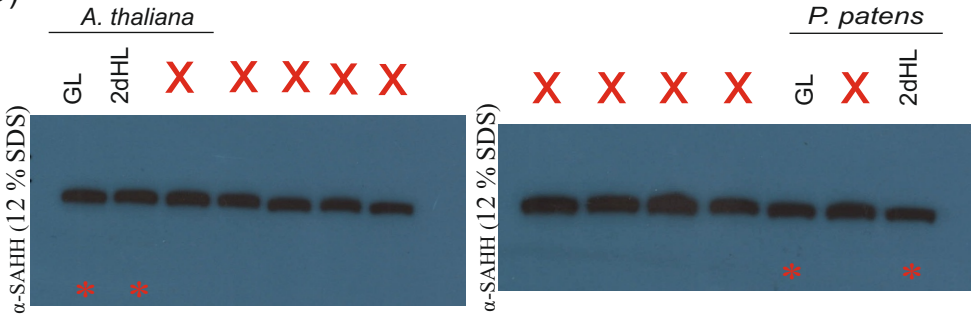

S Figure 1

B)

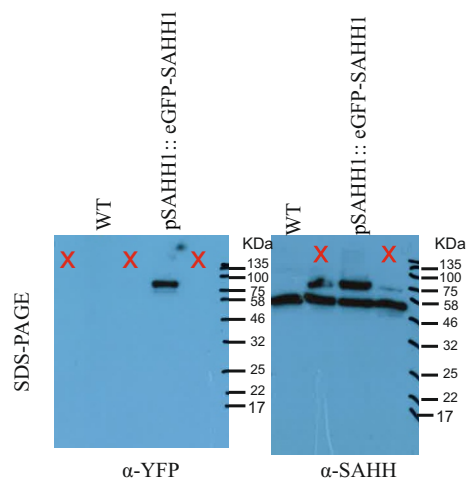

S Figure 2

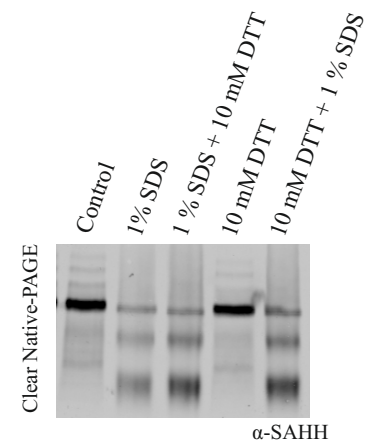

S Figure 3

A)

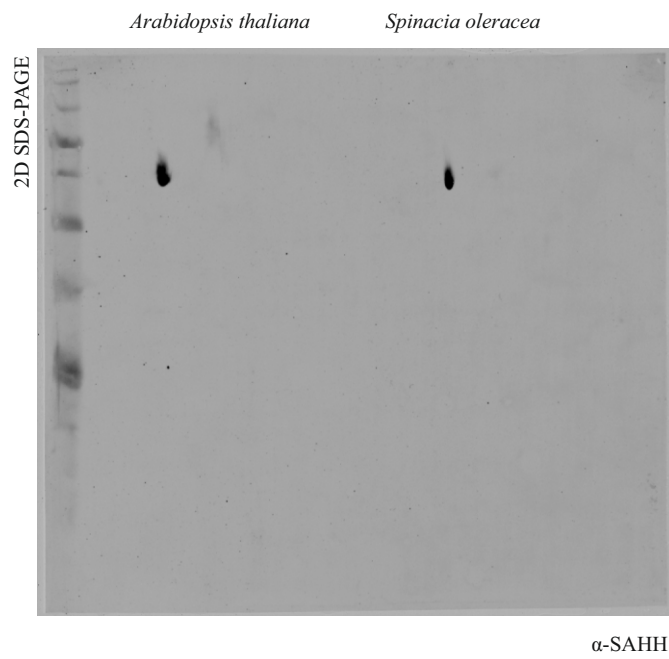

B)

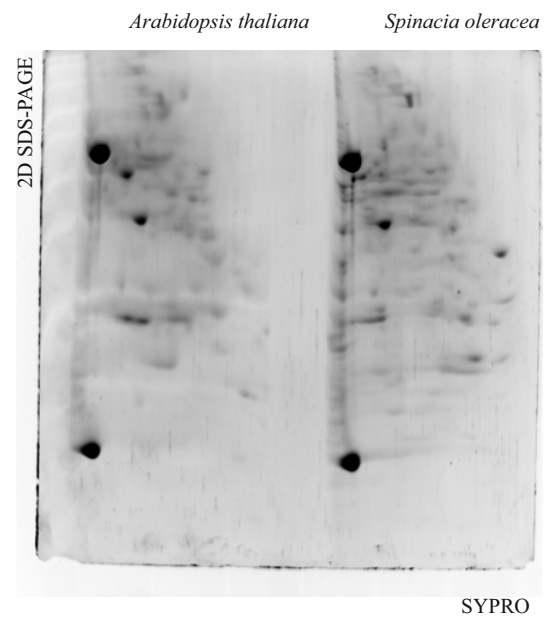

S Figure 4

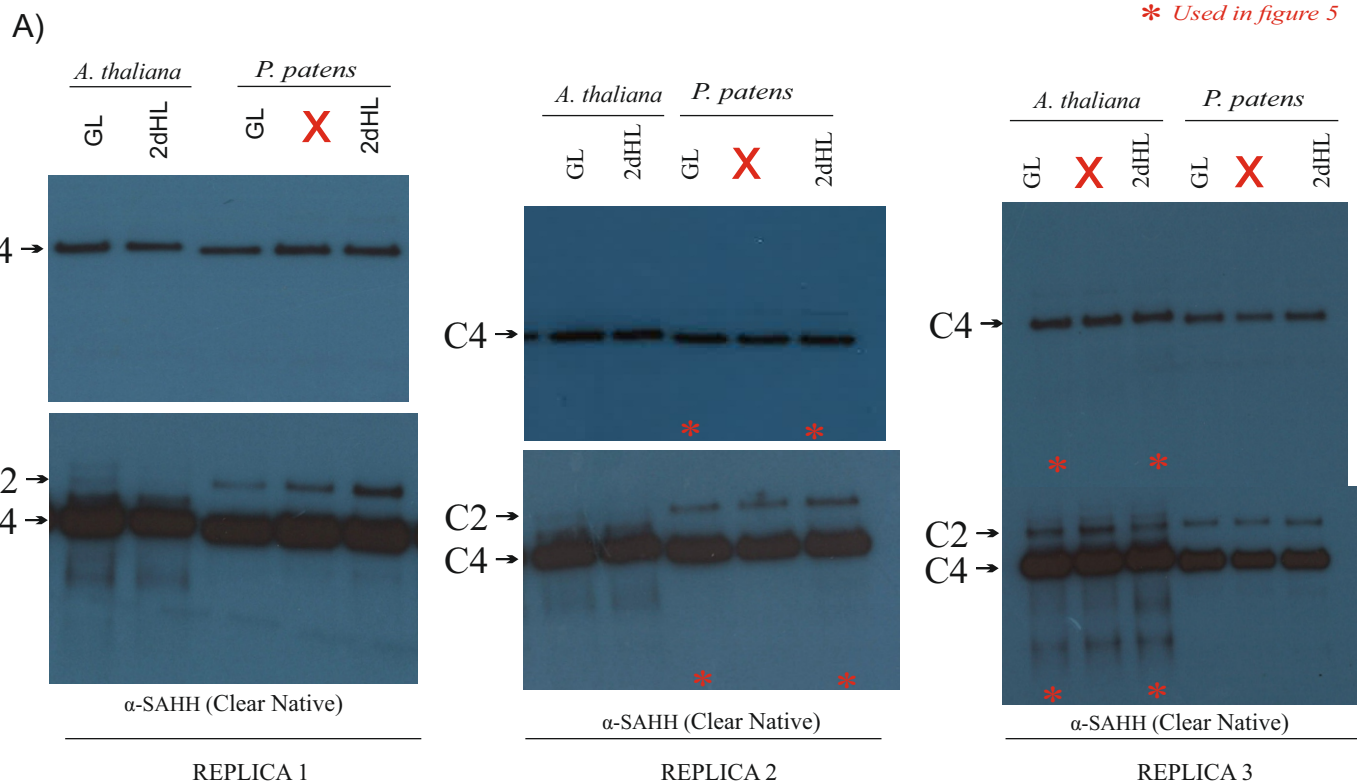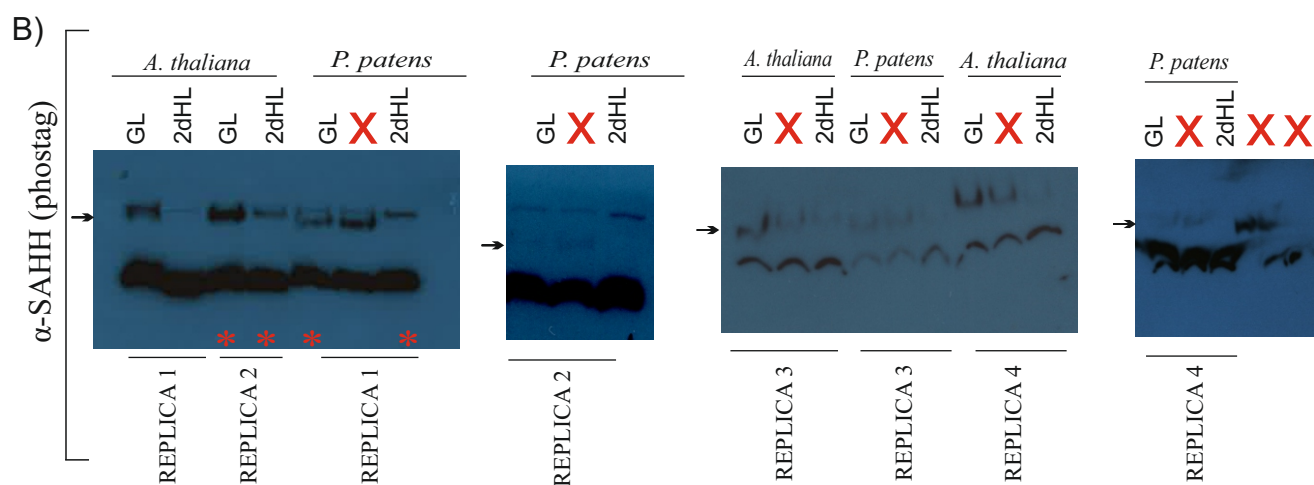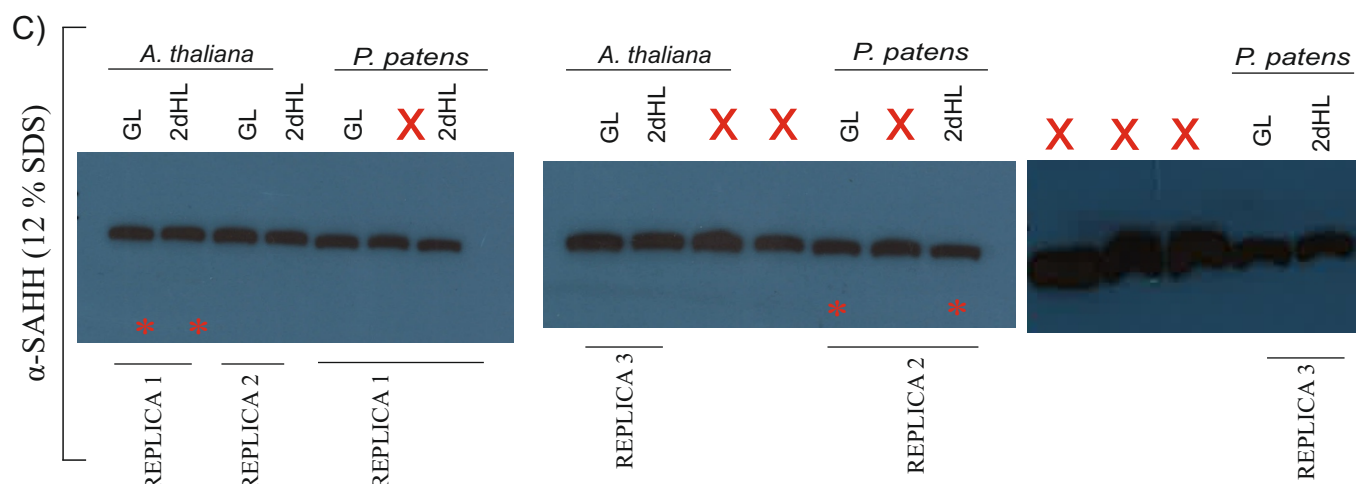

Supplement: S1 Raw images — (PDF) [file pone.0227466.s010.pdf]
