# Supplementary material for: Evolutionary conservation and post-translational control of S-adenosyl-L-homocysteine hydrolase in land plants
Source: PLoS One. 2020 Jul 17;15(7):e0227466. doi: 10.1371/journal.pone.0227466 (PMC7367456; doi:10.1371/journal.pone.0227466)
Supplement: Attachment — Submitted filename: Response_to_reviewers.docx [file pone.0227466.s012.docx]

Dear Reviewers,

Thank you very much for your very helpful critical comments to our revised manuscript “Evolutionary conservation and multilevel post-translational control of S-adenosyl-homocysteine-Hydrolase in land plants” (PONE-D-19-35012R1).

Below we present our responses to the specific points made by each reviewer.

We hope the new version would meet your criticisms and the PLOS ONE’s criteria for publication.

Sincerely,

Saijaliisa Kangasjärvi

**Reviewer #1:** The Authors have addressed my comments. Therefore, the present manuscript can be accepted for publication. I have identified a few of remaining concerns/minor corrections that that also need to be made in a final revision:
1. Page 4, line 93: is “in vivo”, should be: “in vitro”.
2. Page 18, Table 1: It is sufficient to report identity/similarity values with two - three significant digit after the period.

- Thank you for your time to go through this work again, and for noticing these mistakes. They have now been corrected.

**Reviewer #2:** The authors seemed to have ignored most of the comments of the reviewers. We have all asked for major revisions, and yet none of the main figures were modified. Moreover, some of their answers have brought up new problems. The manuscript still appears very rough and premature.

There is still only on replicate shown in Fig. 5. If the authors have really performed the experiments three times as they now specify, there should be no problem showing in sup info the other two replicates. Ideally, though, as I previously asked, to have 2 or 3 replicates lanes (from different biological samples) for each treatment would make the manuscript much more believable.

The statistical test used in Fig S4 is apparently written to be t-test. This is not appropriate as changes in C2 and C4 for one species are not independent (same lane) and should be analysed together.
There is no significance shown for C2 in S4 (C2 label is missing, by the way) despite more pronounced differences in bar heights, does it mean it was not significant or the significance label was also forgotten? The text does not really make it clear either. Which changes were significant? Quantification of gel lanes from Fig. 5 and statistical analyses shown in Fig. S4 are actually very important and belong to the main figure, especially since light-dependent changes in SAHH complex are mentioned in the abstract.

- Thank you for your time to make a second assessment. We have now further improved the presentation of the results presented in Fig. 5.

- Because showing all the replicates takes quite a considerable amount of space, we would very much prefer to present the independent biological replicates of all the blots as a new Supplemental Figure (S4 Fig). The uncropped images used to construct Fig 5 and S4 Fig have also been added in the raw data file.

- Quantification of C2 and C4 abundance is now included in the main Fig. 5, and the missing label has been added. The statistical test to assess the data has indeed been a t-test. This is because we have to quantify the C2 and C4 abundances from differently exposed films; when C2 can be visualized, C4 is already over-exposed on the film. For this reason, we also show the shorter exposure time for C4 in Fig. 5 and S4 Fig, and feel that the statistics for C2 and C4 abundance should be handled independently from each other. The significance levels are now clearly indicated in the figure legend, and we have also modified the text accordingly in the results section, on pages 19-20 and in the discussion on page 25. Also the abstract has been modified for consistency. In addition, we replaced the P. patens CN-gel picture in Fig. 5A with Replicate 2, since it corresponds better to the data obtained by quantification of the SAHH complex abundance.

- We also changed Fig. 5B to allow better comparison of the qualitative changes observed on Phostag gels: the Arabidopsis blot was replaced by a different replicate (Replica 2 on S4 Fig), which was run in parallel with Physcomitrella samples on the same gel.

The authors now seem to have used MS to identify spots shown in S3, but S3 just shows a picture of a 2D gel without any annotations on which spots were analysed to confirm the presence of SAHH. The authors should be precise about what was analysed (which 2D gel, which spot(s)) this time by MS.

- Thank you for this comment. We have now improved the text (Page 18) and the S3 figure legend to clarify the message. We mapped the main SAHH protein spot (corresponding to complex 4) on the 2D gels in Arabidopsis and spinach by immunoblotting with SAHH antibody. Because we were interested in demonstrating that the detected “complex 4” contains SAHH also in spinach, only those spots were excised from the 2D gels of Arabidopsis and spinach samples and analysed by MS. The excised spots are now indicated as “SAHH” on S3 Fig and the identified proteins are listed in the two sheets of the Table S3. Identified SAHH isoforms are now highlighted with yellow in the table.

While the discussion sometimes goes well beyond what is actually shown in the manuscript, there is not discussion about the contribution of SAHH1 and SAHH2 in the protein complexes the authors describe. Does their antibody also recognise SAHH2? If not, why not? In mammals Ahcyl1/Ahcyl2 interacts with Ahcy and so presumably also in plants. The authors indeed mention SAHH2 in the introduction, but then completely forget about it in the results and the discussion.
If the authors really have used MS, they should have been able to differentiate between SAHH1 and SAHH2. Why not?

- This are also good points. We have now made some small edits in the discussion. Also, we indeed typically identify both SAHH1 and SAHH2 in the spot samples we analyse by MS, and both isoforms are also present among the identifications in the sheet for Arabidopsis in the S3 Table. Due to the very high sequence similarity between SAHH1 and SAHH2, the antibody also very likely recognizes both isoforms. It is therefore very hard to assess their relative proportions in the oligomeric complexes. We have now addressed this issue better in the revised version of the manuscript, on pages 4, 13 and 18, and in the discussion. Also, the SAHH isoforms have been highlighted in S3 Table.

Really, I can't recommend this manuscript for publication at the moment.

- We hope the manuscript has now been sufficiently improved.

Reviewer #3: Most of reviewers' comments were properly corrected and revised according to reviewers' points and suggestions. But I still feel few parts of the manuscript were not clear because of data quality. So I would like to tell authors one thing that authors need to prepare your data clearer next time.
Anyhow, I recommend the paper for publication.

- Thank you for the advice and the recommendation.
